# Developing a web-based information resource for palliative care: an action-research inspired approach

**DOI:** 10.1186/1472-6947-7-26

**Published:** 2007-09-14

**Authors:** Annette F Street, Kathleen Swift, Merilyn Annells, Roger Woodruff, Terry Gliddon, Anne Oakley, Goetz Ottman

**Affiliations:** 1 Clinical School of Nursing, La Trobe University/Austin Health, Lv 4 Austin Tower, Heidelberg VIC 3084, Australia; 2 Nursing & Midwifery, La Trobe University, Bundoora VIC 3083, Australia; 3 Director of Palliative Care Austin Health, Suit 9, 210 Burgundy Street, Heidelberg VIC 3084, Australia; 4 Research and Development, Royal District Nursing Services, 31 Alma Rd, St. Kilda VIC 3182, Australia; 5 CEO, Melbourne City Mission, 471 Nicholson St., North Fitzroy VIC 3068, Australia

## Abstract

**Background:**

General Practitioners and community nurses rely on easily accessible, evidence-based online information to guide practice. To date, the methods that underpin the scoping of user-identified online information needs in palliative care have remained under-explored. This paper describes the benefits and challenges of a collaborative approach involving users and experts that informed the first stage of the development of a palliative care website [1].

**Method:**

The action research-inspired methodology included a panel assessment of an existing palliative care website based in Victoria, Australia; a pre-development survey (n = 197) scoping potential audiences and palliative care information needs; working parties conducting a needs analysis about necessary information content for a redeveloped website targeting health professionals and caregivers/patients; an iterative evaluation process involving users and experts; as well as a final evaluation survey (n = 166).

**Results:**

Involving users in the identification of content and links for a palliative care website is time-consuming and requires initial resources, strong networking skills and commitment. However, user participation provided crucial information that led to the widened the scope of the website audience and guided the development and testing of the website. The needs analysis underpinning the project suggests that palliative care peak bodies need to address three distinct audiences (clinicians, allied health professionals as well as patients and their caregivers).

**Conclusion:**

Web developers should pay close attention to the content, language, and accessibility needs of these groups. Given the substantial cost associated with the maintenance of authoritative health information sites, the paper proposes a more collaborative development in which users can be engaged in the definition of content to ensure relevance and responsiveness, and to eliminate unnecessary detail. Access to volunteer networks forms an integral part of such an approach.

## Background

Several recent reports tabled by government as well as non-governmental health agencies foreground a significant disparity between the information needs of health professionals, patients and caregivers givers and the availability of such information. In response, the reports urge governments to invest in the information, education, and training resources available to health-care professionals, patients and caregivers, as well as the wider community, in order to foster awareness and capacity regarding palliative care [2-10].

Whereas the increasing demand for consumer health information on the Internet has been also the topic of much recent medical, social science and information science literature [11-22], there has been a noticeable absence of studies that focus on how to adequately design such online resources. This paper describes the advantages and challenges of a collaborative, action research-inspired approach involving users (doctors, nurses, and consumer and caregiver representatives) that informed the development of a palliative care website [1] providing accessible information for the general public, along with evidence-based pain, symptom and psycho-social information for health professionals.

The aim of the government-funded study was to improve the existing website of Palliative Care Victoria (PCV), a state-level palliative care organisation. In light of the large volume of inquiries from community doctors, nurses and members of the general public, the Director of PCV was of the view that the organisation's response to such enquiries could be improved by enhancing the information for the general public, along with evidence-based pain, symptom and psycho-social information for health professionals, in particular general practitioners and community nurses available through its website. The study also took into account more global policy issues. For instance, it took into account the key recommendations outlined by the Improving Supportive and Palliative Care for Adults with Cancer report tabled by the United Kingdom-based National Institute for Clinical Excellence in 2004. Among other the report underscored the value of high quality information for patients and caregivers, the importance of ensuring that the views of patients and caregivers are taken into account in developing and evaluating cancer and palliative care services, and that patients and caregivers have easy access to a range of easy to read, high quality information materials about cancer and palliative care services that are provided free of charge [27]. Yet, the study's main aim was to tackle a range of information gaps highlighted in recent national government reports [3,4,6,7]. In particular, the study responded to the policies outlined in the Australian Government National Palliative Care Priorities, namely to improve professional awareness and commitment of health professionals to the role of palliative care practices, and to provide quality information to patients and their caregivers [23,24].

## Method

**Figure 1:**
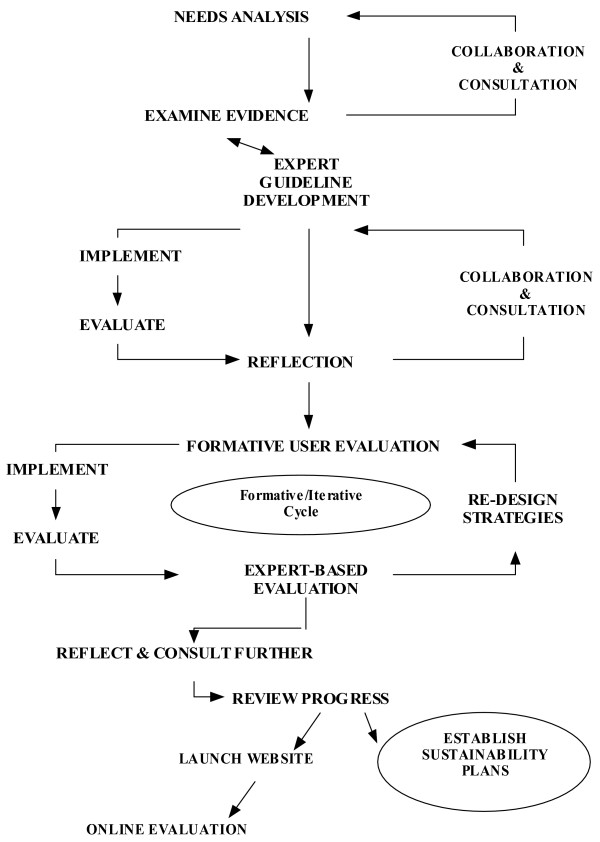
Action Research Process.

As the project was designed to be responsive to feedback from potential user groups an action research approach was undertaken. The actual research design and reporting structure was negotiated with the funding agency at the outset. The action research process enabled the project staff to design an iterative process of continuous improvement through trial, analysis, and feedback from key stakeholders in order to adapt and modify the project to meet emergent needs [25-30]. In this project, the study of effects and outcomes of one stage informed the development process of the next stage and provided guidance for the development of the investigative framework that best addressed the evaluation questions. The following Figure (1) provides an overview of the action research methodology employed.

Theoretical contributions to the field of educational multi-media design indicate that issues regarding content, language, and 'look and feel' could potentially be resolved through an ongoing consultation with expert user groups [31-33]. In fact, it is widely acknowledged that an ideal design process of educational online resources is based on cybernetic principles of general system theory, which relies on cycles of self-testing, ongoing feedback, and constant adjustments [34]. However, this extremely flexible and responsive approach to web design, although able to accommodate changes in and unanticipated effects of the implementation progresses, is seldom employed due to the high cost of cycling back to stages previously completed [34]. In this project, we employed an iterative, formative development process inspired by these principles.

User feedback was collected in the form of focus group discussions, summary grids, written comments, and 'think aloud protocols', a method where users navigate the prototype site delivering verbal comments throughout the process [34]. An audit trail was designed to keep track of user responses.

**Figure 2:**
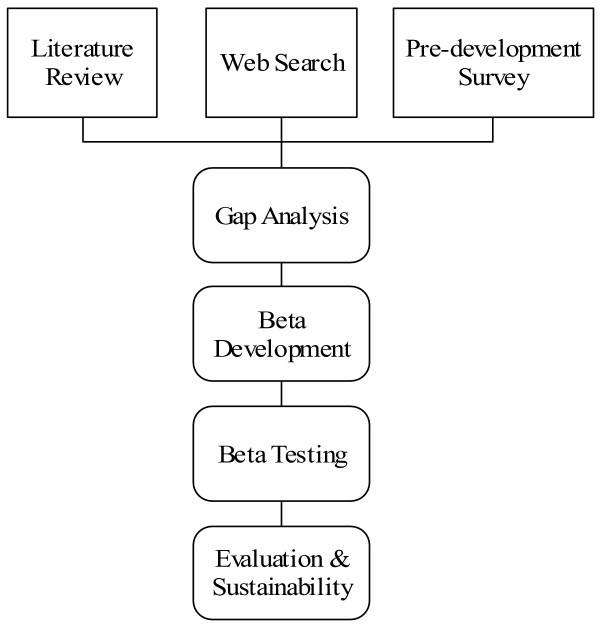
Main Project Stages.

The project was constructed in four main stages that were implemented between March 2003 and May 2005. The pre-development stage involved identification of the needs of the target audiences through the literature review, a pre-development online survey, and a gap analysis undertaken by the User Working Groups (UWGs) made up of consumers and health professionals under the oversight of the Project Reference Group (PRG) of key stakeholders. After a lengthy consultation and development process, a test website (beta site) was developed by the Melbourne-based Centre for Online Media and Educational Technology (COMET) for eventual transfer to the Palliative Care Victoria website. The beta (test) site development was undertaken with input from external experts and the User Working Groups. The beta site was then assessed by key stakeholders as well as consumers. Finally, as last modifications were made to the site the sustainability plan was implemented in conjunction with PVC. The following Figure (2) gives an overview of the major project stages:

Because our aim was to include, as much as possible, the views and expertise of all potential users of the website, it was attempted to include as many representatives of potential stakeholder groups as possible in the PRG and UWG so that they would be adequately represented during the various project cycles.

### Stage 1 – literature review, pre-development survey, website search

The thematic literature review was based on an extensive search of electronic databases (Medline, CINAHL, PubMed) as well as web-based searches (Google, Google Scholar) for additional material, such as reports commissioned by government as well as non-governmental agencies. Due to the limited scope of the literature on palliative care, sources dealing with online cancer information were included in this literature review. Permutations of search terms (Palliative, Cancer, Care, Online, Internet, Information, Needs) were used to collect a total of 388 bibliographical entries in an EndNote file. A researcher scanned the abstracts of these articles for relevance, thus eliminating 314 articles. The remaining 74 articles were sourced for inclusion into the literature review. In addition a separate literature review was conducted focusing on the available clinical evidence in subcategories of palliative care such as pain, medication, and psychosocial needs. For the clinical subject areas systematic reviews submitted to the Cochrane database, clinical guidelines, as well as expert consensus panels were included. The research underpinning the clinical information included on the website was evaluated and ranked by clinical palliative care experts. Due to the dearth of research-based evidence in sub-categories such as psycho-social needs, the PRG decided to include also what is considered 'best practice' in those fields.

A short online survey was designed to provide a cursory overview of users and their palliative care information needs. The 10 question survey was posted on the existing PCV website. Participants were asked:

• why they accessed the website

• whether they were health professionals or patients/caregivers/members of the public,

• what kind of information they were looking for,

• whether they found the desired information,

• to indicate areas that should be considered for inclusion in the website,

• whether it was easy to locate the information on the existing site, and

• to comment information gaps in the current site.

Internet users who accessed the site were asked to complete the form online. The survey was posted on the internet for a total of five and a half months beginning from 8 October 2003. Response to the survey was anonymous. The webmaster collected the responses and coded and pre-compiled the data. Subsequently, the responses were screened, duplicates or void forms were eliminated, and the pre-compiled data was analysed.

Furthermore, an extensive search of existing health websites was undertaken to identify the key features of reputable or 'Health on the Net' (HON) accredited websites. The Economic and Social Council of the United Nations accredited HON Foundation is the leading organization promoting and guiding the deployment of useful and reliable online medical and health information, and its appropriate and efficient use.

### Stage 2 – gap analysis

Taking into account the preliminary findings from the literature review, the web search, and the results of the pre-development survey, both UWGs drafted a detailed gap analysis framework addressing the information needs of the stakeholders they represented. Moreover, the Project Reference Group as well as key stakeholders and the experts from the Centre for Online Multimedia and Educational Technologies (COMET) at La Trobe University, evaluated the original website of Palliative Care Victoria for content, relevance, accessibility, links, search potential, design features and language level. The original website catered predominantly for palliative care 'insiders' (mainly general practitioners and physicians) and posted a series of pamphlets and press releases that could be handed on to patients. The literature reviews in conjunction with the website search as well as guidelines provided an overall framework for content and design, whereas the online survey gave an initial indication of the kind of subject areas health professionals, patients, and caregivers might expect the future site to cover. In this sense, the 'gap analysis' provided very rough guidelines for the construction of a beta site. However, it is important to remember that, because of the formative, iterative process that underpinned the design of the website, needs and gap analyses were ongoing as stakeholder representatives co-designed and debated the content of the site through the numerous iteration cycles.

### Stages 3 and 4 – beta development and testing

An effective Project Reference Group was formed. The **Project Reference Group **(PRG) brought together nine professionals active in a range of palliative care-related fields and who held senior positions in councils, associations, and academia. Moreover, the PRG included a Project Officer who had extensive knowledge of the palliative care field. The Project Officer moderated most group discussions. The sizeable networks of these individuals were instrumental during the implementation phase of the project as many of the User Working Group members were recruited through these networks. Moreover, these participants brought to the project expertise in clinical palliative care, project management, team leadership, online educational technology, and research/evaluation techniques that could be drawn upon over the course of the project. Several members of the PRG joined an editorial panel that took responsibility for drafting editorial guidelines and monitoring content. The PRG functioned as the central mediation vehicle and most editorial decisions were discussed within this forum.

Two **User Working Groups (UWG) **were formed to participate in the sourcing and evaluation of content. One UWG drew on the expertise of focus group participants brought together by the Cancer Information & Support Services (CISS) run by The Cancer Council Victoria, as part of their activities directed at identifying unmet needs for people affected by cancer. The working group included 11 cancer patients, caregivers and volunteers. The other UWG was made up of 12 health professionals and included four general practitioners, two palliative care physicians, and six palliative care nurses representing nine organisations. Again the networks associated with these members were very extensive and greatly facilitated the user-based evaluation of the process. It was envisaged that these groups would meet on a monthly basis. UWG participants as well as members of their professional networks were asked to provide feedback in the form of summary grids (see additional file 1) after each design cycle.

In addition to the PRG and UWGs, a substantial number of experts in fields such as palliative care, nursing, community support, or allied health made major contributions to the project. Also, a consumer focus group was conducted by a market research firm. This focus group dealt primarily with the look and feel (colour schemes, navigability) of the website. Because representatives of all major stakeholders were involved in the reference and working groups, it is reasonable to suggest that these stake holder groups had a direct input into the design of the web site. Moreover, bearing in mind that 197 and 166 participants respectively responded to the pre-development and evaluation online surveys, it is likely that over 300 individuals contributed to the design and evaluation of the PCV website.

### Stage 5 – evaluation

An evaluation survey was attached to the new website. Not withstanding minor changes, the evaluation survey mirrored the content of the pre-development survey. Based on the experience with the pre-development survey, it was decided to separate the information for the public and health professionals. The pre-development survey did not make this separation. Moreover, the evaluation survey was more structured providing a list of possible responses that participants could tick in addition to an 'other' category followed by an open-ended prompt to specify this response.

## Results

### Stage 1 – literature review and online survey

The literature review revealed a growing recognition of the existence of unmet information needs regarding palliative care practice [6,9,35-44]. Recent evidence suggests that doctors, nurses, patients, and caregivers increasingly turn to the internet to meet some of these information needs [12-14,16,18,20,22,45-51]. One of the key concerns that surfaced in the literature focusing on online health is the issue of quality. A large number of studies express concerns with regard to the quality of information provided by web sites that post cancer health information on the Internet [52-58]. Most of these studies alert to the fact that websites commonly display misleading, incomplete, and/or erroneous information and that the confidentiality of data is often not assured [55,59-64]. Surveys of websites and the way users access them suggest that it is difficult and time consuming to identify good online resources and that the capacity of users to find and access valuable information is limited [65,66]. For most researchers the key problem faced by users is how to evaluate the quality of online information and how to establish the trustworthiness of sources.

In particular, researchers [4,6,7,57,64,66-72] identify the following key issues:

• Lack of editorial control,

• misleading or incomplete content,

• inappropriate design,

• inappropriate cultural bias,

• inappropriate commercial content,

• lack of readability (especially for cultural minorities or people with disabilities),

• inappropriate navigation tools,

• overall disorganisation of the site,

• accessibility of audio and visual resources, and

• problems regarding volume management and maintenance.

In response to these quality concerns, authors propose the use of tools to evaluate the quality of health information web sites [73,74], and/or to introduce obligatory standards or evidence-based editorial criteria to improve the quality of health information sites [54,66,75,76]. Yet, recent contributions to the field have been critical of evaluation tools, claiming that they are largely ineffective [77].

Other commentators have recommended the design of designated portal sites or gateway servers that organise and edit web content [78]. To some degree, sites such as OncoLink, the Cancer Net, Cancer Help UK, and others fit into this category. A number of articles give an overview of [79-84] and/or evaluate such sites [85,86]. Designated portal sites or gateway servers generally enjoy impressive approval ratings among clinicians as well as the general public. Nevertheless, recent articles have raised the issue of sustainability and costs, update, and maintenance work faced by online health information providers [87]. More recent evidence questions the proposition that the design of web portals lastingly improves the quality of online health information [88].

In summary, the literature suggests a need for a more comprehensive information and education strategy focusing on palliative care. It makes it clear that an understanding of the audiences to be targeted as well as their online information needs is crucial for the development of reliable and effective health information websites [22]. Moreover, it appears that for online health information resources to be efficient, four basic conditions have to be met: content needs to be clinically authoritative, accessible, sustainable, and has to respond to the information needs of potential user groups. Furthermore, the design process benefits from an ongoing involvement of stakeholder groups. However, there is a dearth of evidence regarding the information that should be included in a site providing educational information on palliative care to health professionals as well as to patients and associated caregivers as well as the methodology might be successfully employed to that end.

### Pre-development survey

The survey attached to the existing website generated a total of 197 responses (see Table 1 below). All responses were considered valid, although not all questionnaires were completed in full. The majority of respondents who did not complete all ten questions left out the open ended questions. One hundred and thirty two (67.01%) respondents identified themselves as health professionals and 63 (31.98%) as non-health professionals.

**Table 1:** Responses to Pre- and Post-Development Surveys

	**Existing Website**	**New Website**
Period of Survey online	Oct 2003 – March 2004	May 2006 – March 2007
Responses (n=)	197	166
Health Professionals	132 (67%)	121 (73%)
Caregivers/Patients/Public	63 (32%)	45 (27%)
What information were you looking for on the PCV website?	*Public: * • To find information that would assist me in my care giving role (15%) *Health Professionals: * • To find information in support of professional practice (79%) • To find information that would assist patients in their care-giving role (15) • Clinical aspects of professional practice (13%) *Public & Health Professionals: * • To find resources on palliative care (66%)	*Public: * • To understand the health care system better (96%) • About palliative care services in Victoria (40%) • About helping someone who has an illness (33%) • To help the person I care for to receive the best possible treatment (31%) • To help me deal with my feelings (27%) • To understand what palliative care is (26%) • About living with my illness (11%) • About palliative care services in Australia (11%) • To help me put my affairs in order (9%) • To help me receive the best care possible (4%) *Health Professionals: * • Education available in palliative care (50%) • Symptom Management (37%) • Advance Care Planning (31%) • Loss and Grief (25%) • Talking with clients and their families (23%) • Palliative care services in Victoria (22%) • Palliative Care Victoria (21%) • Death (21%) • Services available to my clients (21%) • A website clients and their families could use as a resource (17%) • The meaning of palliative care (13%) • Palliative care services in Australia (4%)
Did you find the information you wanted?	128 yes (65%) 68 no (35%)	137 yes/some but no all (82%) 18 no (11%)
Was it easy to find the information?	130 yes (66%) 57 no (29%)	122 yes (74%) 38 no (23%)
Additional information to be included:	• Professional Practice • Grief • Advance Care Planning • Volunteering • Pain • Jobs • Ethics • Home Care • Contact Details	*Health Professionals: * • Courses/Workshops/Conferences • Scholarships • Careers *Public: * • List of places to go as a family • Sings of dying/clinical information • Volunteering • Jobs • Financial entitlements
Comments about the website	• the need for better navigation tools, • the site's limited content, • the ineffectiveness of the site's search engine, and • the fact that the site had not been updated.	• Plain, clear and unemotional, but still sensitive – well done. • Well-mapped out, easy to navigate.

The most important reasons respondents provided for accessing the existing palliative care website were to seek information in support of their role as health professionals (79.26%) and professional practice (13%), to find available resources (66.21%), and to find information that would assist them in their care-giving role (15%). Of the respondents 128 (64.97%) indicated that they had found the information they were looking for and 68 (34.52%) stated that they did not. Respondents to the existing website thought that in-depth information about topics such as pain, grief, volunteering and ethical issues should be included. Also noted was the absence of links for volunteers, a site map, a list of recommended publications, a listing of employment opportunities and short courses, comprehensive information for professionals, course information on professional practice, and contact details.

When asked if it was easy to find the information that they were seeking, 130 (65.99%) of the respondents to the existing site gave an affirmative response, whereas 57 (28.93%) responded negatively. Sixty two (31.47%) participants specified their answer. Of these, 26 (13.20%) gave positive feedback, whereas 20 (10.15%) provided constructive criticism. The issues raised by these respondents were:

• the need for better navigation tools,

• the site's limited content,

• the ineffectiveness of the site's search engine, and

• the fact that the site had not been updated.

### Stage 2 – gap analysis of existing site

The expert panel discovered limitations in the organisation, content, language, accessibility, design, navigability, and readability of the existing PCV website. To some degree this finding was expected, given the limited resources available for the ongoing support and development of a site mainly administered by volunteers. Among the most important shortcomings noted by the panel was the very limited scope of the website, the lack of effective search capacity, the complexity of the language used and accessibility issues for people with reading difficulties. It was felt that the website was largely providing information to 'insiders' in the palliative care community but not to general health professionals nor to a substantial segment of caregivers, patients, and the general public.

The two working parties considered material from the gap analysis, the website search, as well as a preliminary analysis of emerging survey responses. Also the working party members' opinions of unmet needs as identified from their own experience or from interaction with their peer networks. Discussion ensued about how much information should be available for a quick scan approach, how much should be printable information sheets or pamphlets, and what links should be included to provide greater depth of knowledge. Each working party developed a framework for the health professional or the consumer web portal respectively. An overview of these frameworks is provided in Tables 2 and 3.

**Table 2:** Framework for health professional portal

**The principles & practice of palliative care**	What is palliative care – definitions	
	Communication the corner stone of palliative care	Building a supportive relationship
		Providing information
		Communicating bad news
		Involving family/caregivers
		Working and communicating within the multidisciplinary team.
	Ethics	
	Frequently asked questions	
**Symptom care**	Pain	About Pain
		Understanding pain
		Assessing pain
		Treating pain
		Barriers to pain control
		References
	Respiratory symptoms	Breathlessness
		Cough
		Terminal respiratory congestion
	Gastrointestinal symptoms	Nausea and vomiting
		Bowel obstruction
		Constipation
	Constitutional symptoms	Anorexia
		Weight loss
		Weakness and fatigue
	Neurological symptoms	Acute confusion and delirium
		Terminal restlessness

**Supportive care**	Advance care planning	What is Advanced Care Planning?
		Talking about Prognosis and usual course of disease
		Advising the patient for Future Decision Making
		The Medical Treatment Act and refusal of Treatment
		Wills
		Funerals
		Genetic counselling
	Loss Anxiety & depression	Normal reactions to loss of good health
		Complicated reactions to loss of health
	Changing circumstances	Changing family/relationship roles
		Sexuality and Intimacy
		Spirituality
	The family/support network	Information and Support
		Respite and Education Services
	What needs to be done when the patient dies	For Family/Caregivers
		Health Care Professional Care
		Medical care

**Grief and bereavement**	Normal reaction to loss of another	
	Resources for helping the bereaved	
	Complicated grief & bereavement	
	Medication & grief	

**Table 3:** Framework for consumer portal

Categories	Content	To Cover
About Palliative Care	What is palliative care?	Terminal illness Precious time Maximising opportunities in life FAQ
	Who provides palliative care?	Health care team Eg. pastoral care, family as the key unit provider
	How do you access palliative care?	Self referral GP Hospital physician Community services Specialist PC services
	When does palliative care begin?	When required
	When can I ask about pain relief and symptom control?	When do you discuss palliative care and supportive issues?

Navigating the Health Care System	Palliative care and the health care system	Feeling abandoned? Move from the acute serviced orientation Home or Hospital ?
	Talking to your doctor	Hints and strategies
	Talking with other health care professionals	Hints and strategies
	Complementary therapies	Types Benefits autions
	Unhappy with the health care system?	Patient rights Avenues for mediation

Receiving palliative care	Planning for the time you have	What we know about dealing with a life limiting diagnosis
	Managing your symptoms	Fatigue Frustration Pain Difficulty breathing
	Managing your life	Living with uncertainty Have your affairs in order
	What to expect when you are dying	Fears, pain, reaction and behaviours of others
	Creating your memory	Healing old wounds Leaving a message Giving treasures to be treasured
	What others have done	Stories & Obituaries

Caring for someone receiving palliative care	Being a caregiver	The role/Rights/Support Resources
	Understanding your situation	Physical and psychological demands Personal loss & growth Dealing with different coping strategies Need for a break
	Creating memories	Events/Diary/Photos The stories of other caregivers
	Obtain important information	Deciding what is important
	Physical aspects of caring	Bed comfort/Nutrition Medications/Hygiene
	Recognising and managing symptoms	Fatigue/Pain Lack of appetite or urinary control

Caring for someone who is receiving palliative care cont'd	When death occurs	What to expect when the one you are caring for is dying Letting go What to do when someone dies Caring for the body after death

Moreover, working parties in conjunction with the PRG defined the overall scope of the new website delimiting the topics that would be covered by the site. Excluded from the site were topic such as careers and financial entitlements.

### Stage 3 and 4 – beta development and testing

Because this phase has been published in detail elsewhere only a short summary of its key elements of this phase is given. The formative user evaluation underpinning the project worked exceptionally well. Perhaps the most surprising element of this phase was the level of support that participants were willing to invest in the project. Whereas the UWGs provided crucial information during the initial stages of the project, the editorial panel in conjunction with key user groups associated with the representatives participating in the UWGs provided valuable input during the evaluation of the beta test site. As anticipated, feedback occurred continuously throughout the project and was circulated through the PRG and UWGs. As a result, the PRG and UWGs were able to respond quickly to user feedback and shape the content and overall design of the website accordingly. Expert evaluators became important mediators of conflicts. Referring to 'good practice' guidelines and by providing their 'expert opinion', they, supported by the PRG, managed to resolve diverging views as well as conflicts of interests that surfaced within UWGs.

### Stage 5 – evaluation survey

The online survey posted on the new website resulted in a total of 166 responses (see Table 1 below). A total of 121 (73%) respondents identified themselves as health professionals and 45 (27%) as care givers, patients, or members of the public.

Care givers, patients, and members of the public responding to the new website sought primarily information regarding the health care system (96%), about palliative care services in Victoria (40%), as well as information in order to help others with an illness (33%), help the person they cared for (31%), and help them cope (27%). Health professionals responding to the new website (B) sought information about education (50%), symptom management (37%), advance care planning (31%), loss and grief (25%), talking with clients and families (23%), Palliative Care Victoria (22%), death (21%), services available to their clients (21%), and a resource website for clients (17%).

Of the respondents, 137 (82%) indicated that they had found all or some of the desired information and 18 (11%) stated that they did not. Respondents of the evaluation survey thought that topics such as further education, conferences, scholarships, as well as lists of places to go to as a family, signs of dying/clinical information, volunteering and financial entitlements should be considered.

A total of 122 (74%) respondents claimed that it was easy to access the desired information, whereas 38 (23%) claimed that it was not.

Comments focusing on the new website were positive regarding content and design.

## Discussion

Involving users in the identification of content and links for a palliative care website as well as the evaluation of the site's 'look and feel' is time-consuming and requires initial resources, strong networking skills, and ongoing commitment. However, the benefits of engaging key stakeholders in defining and refining the scope and content of such a website outweigh the disadvantages. Over the course of this study, user involvement led to the widening of the scope of the website and alerted the project group to shortcomings regarding content, language, presentation, and usability.

The original intention of the expert re-developed website was to provide information to the consumers and for health professionals in the community who did not have continual access to palliative care expertise. User involvement significantly augmented these aims. In addition to the original intention, the stakeholder-conducted gap analysis in conjunction with the user online survey identified the need for a site that

• provided reputable expert or evidence-based information,

• addressed information requirements in areas such as professional practice, ethical dilemmas or practice standards, as well as topics related to managing palliative pain, grief, advanced care planning, or home care,

• provided clinical content in accessible lay language for the general public,

• guided consumers through the health care system

• provided tools to enhance medical and consumer decision making,

• supplied printable pamphlets for patients and caregivers,

• regularly monitored and updated links and material,

• provided key points that can be easily visually scanned, and

• does not contain distracting graphics that increase download time.

These points would have been missed if the project had proceeded with a common, expert-based web-design.

To some extent, the success effect of the iterative, participatory design of the website is visible in the evaluation survey posted to the new website. For instance, a comparison of the pre-development survey with the evaluation survey shows a decrease of visitors that did not find the information they were looking for from 35% to 11%. Concurrently, the percentage of participants who thought it easy to find the desired information increased from 66 to 74.

Interestingly, since going live, the new website has become an education resource for nursing staff, students, as well as allied health workers. In fact, 46% of the evaluation survey respondents were nurses. Moreover, the evaluation survey suggests that an, albeit small, but nevertheless important number of care givers and patients is accessing the site. Seventeen percent of evaluation survey respondents were care givers or patients. The overall feedback by health professionals as well as patients and caregivers to the new website was, in contrast to the pre-development survey, exclusively positive. What is more, all of the additional topics that health professional thought might be considered for inclusion were outside the scope of the website as defined by UWGs and the PRG.

Yet, the evaluation survey also demonstrates that the portal for patients, families, and their caregivers might require some finetuning. The evaluation survey suggests that whereas the information needs of health professionals are covered by the new site, this is not entirely the case for patients as well as their families and care givers. Given that the information needs of patients and care givers are likely to be more diverse than those of health professionals, this was expected, however. What is more, only two (1%) of respondents to the evaluation survey were General Practitioners (GPs). The reason for this low share in the overall population of respondents has not been analysed. It has to be born in mind that the evaluation survey reflects only on the first eleven months in the life span of the new website. Future surveys will show whether the site will become a central reference for care givers, patients, and GPs.

What is more, the re-development of the website accompanied by the positive feedback the organisation received has led to significant changes in the way PCA manages information. Whereas the old website functioned mainly as a provider of pamphlets and leaflets on palliative care, the new website has assumed a much more central role in the overall operation of the organisation providing education and information. Moreover, PCA plans to web-cast public relations items on the site. Moreover, encouraged by the re-development of the site, the PCV has applied for additional funding in order to integrate web-based information more fully into its communication strategy. Having become aware of the potential of its online strategy, web-support has assumed a more important role in the organisation. This in conjunction with the additional funding PCV was able to attract has impacted positively on the sustainability of the website.

## Limitations

Several limitations were recorded. Perhaps the most important, and obvious, limitation is the usefulness of the pre-development survey in scoping possible web content for a future potential audience that is not yet accessing the site. Whereas the pre-development survey indicated that more non-health professionals accessed the site than was expected, the pre-development survey gave only limited indication as to the information needs of patients and their care givers. In fact, the development process relied to large extent on the input of the UWG as well as the iterative evaluations to define the portal for non health professionals.

Another limitation that emerged more clearly in connection with subsequent projects is the use of volunteer participants. It is important to bear in mind that not all projects are suited to a development phase based largely on volunteer participation of busy health professionals. Although the overall cost of the project (A$ 184,872) was comparatively low, the study was fairly resource intense. In particular, the study relied on a large number of participants who, over the course of three years, generously donated their time and energy. Clearly, for participants to make such a sustained contribution, they need to be extremely committed. Hence, it is important that web developers bear in mind that the salience of the project outcome is likely to influence the commitment levels and, by extension, the support of participants. Furthermore, because of the need to mobilise all major stakeholders and to involve consumer representatives, this kind of approach requires strong networking skills and only individuals with good links to key user groups will be likely to gain the support of key user representatives.

Also, because of the large number of individuals involved in the project and because of the iterative evaluation cycles, an enormous amount of information will be generated. The administration and processing of this information requires well-honed project management skills. In part this had to do with the ineffectiveness of one of the research tools: the summary grid. The summary grid asked for an assessment of the content of each of the web pages associated with these questions using the following criteria: clarity of information, accuracy of information, usefulness of links, target audience for the page, and layout and further suggestions. This feedback method was time-consuming and only members of the consumer panel and a few members of the health professional panel provided feedback in this manner. However the written feedback was most thorough and provided excellent guidance to adjust language and content, to provide further information, and/or to include further links. However, the majority of participants provided feed-back in an ad-hoc, unstructured manner that did not correspond with the data-collection time points we had set. This generated an extraordinary amount of information that, at times, threatened to overwhelm our project officer. This prompted us to strengthen the methodology of subsequent web design projects involving user participation.

For reasons beyond our control, we were unable to generate a denominator for the responses to the initial and subsequent surveys, as well as for the subsequent utilisation of the website. As a result we are unable to complete the rhetorical loop demonstrating an improved usage of the website. This clearly limits the explanatory power of the study.

## Conclusion

A growing body of literature and government reports highlight the existence of a significant information gap with regard to palliative care. As more and more people use the internet to meet their health information needs, the question of what kind of palliative care information internet users are actually looking for becomes central to any online information strategy. However, research that focuses on the palliative care information needs of internet users is scarce. This study illustrates how a user-based methodology involving key stakeholders and potential users can contribute successfully to the development of content, language, and design of the website as well as to its evaluation. Web developers are encouraged to involve potential users in their website development and pay close attention to the content, language, and accessibility needs of their respective audiences.

## Competing interests

The author(s) declare that they have no competing interests.

## Authors' contributions

AS and KS conceptualised and designed the project. AS, KS, MA, RW, TG, and AO were involved in the data collection effort and provided critical input through the project. AS and GO conducted the final data analyses and data interpretation and drafted the manuscript. MA, RW, TG, and AO revised the manuscript and contributed to its intellectual content. All authors read and approved the final manuscript.

## Pre-publication history

The pre-publication history for this paper can be accessed here:



## Supplementary Material

Additional file 1 Summary Grid Click here for file
