# Supplementary material for: Developing a web-based information resource for palliative care: an action-research inspired approach
Source: BMC Med Inform Decis Mak. 2007 Sep 14;7:26. doi: 10.1186/1472-6947-7-26 (PMC2194759; doi:10.1186/1472-6947-7-26)
Supplement: Additional file 1 — Summary Grid [file 1472-6947-7-26-S1.doc]

**Additional File 1**

**Summary Grid**

| **Section and page topic**  **ABOUT PALLIATIVE CARE** | **Clarity of information** | **Accuracy of information** | **Links** | **How important is this page? Who will read it?** | **Layout** | **Further suggestions** |
| --- | --- | --- | --- | --- | --- | --- |
| What is palliative care? |  |  |  |  |  |  |
| Frequently asked questions |  |  |  |  |  |  |
| Where is palliative care provided? |  |  |  |  |  |  |
| How do I access palliative care? |  |  |  |  |  |  |
| When does palliative care begin? |  |  |  |  |  |  |
| What should I ask about pain and symptom control? |  |  |  |  |  |  |
